# Development and Validation of Aptasensor Based on MnO_2_ for the Detection of Sulfadiazine Residues

**DOI:** 10.3390/bios13060613

**Published:** 2023-06-03

**Authors:** Xiaoling Zheng, Lulan Yang, Qi Sun, Lei Zhang, Tao Le

**Affiliations:** College of Life Sciences, Chongqing Normal University, Chongqing 401331, China; 2021110513064@stu.cqnu.edu.cn (X.Z.); 2020110513031@stu.cqnu.edu.cn (L.Y.); sunqi2017@cqnu.edu.cn (Q.S.); 20132133@cqnu.edu.cn (L.Z.)

**Keywords:** aptamer, MnO_2_, aptasensor, sulfadiazine

## Abstract

The monitoring of sulfadiazine (SDZ) is of great significance for food safety, environmental protection, and human health. In this study, a fluorescent aptasensor based on MnO_2_ and FAM-labeled SDZ aptamer (FAM-SDZ30-1) was developed for the sensitive and selective detection of SDZ in food and environmental samples. MnO_2_ nanosheets adsorbed rapidly to the aptamer through its electrostatic interaction with the base, providing the basis for an ultrasensitive SDZ detection. Molecular dynamics was used to explain the combination of SMZ1S and SMZ. This fluorescent aptasensor exhibited high sensitivity and selectivity with a limit of detection of 3.25 ng/mL and a linear range of 5–40 ng/mL. The recoveries ranged from 87.19% to 109.26% and the coefficients of variation ranged from 3.13% to 13.14%. In addition, the results of the aptasensor showed an excellent correlation with high-performance liquid chromatography (HPLC). Therefore, this aptasensor based on MnO_2_ is a potentially useful methodology for highly sensitive and selective detection of SDZ in foods and environments.

## 1. Introduction

Sulfadiazine (SDZ) is an antibiotic of the sulfonamide family, which is widely applied to prevent and treat bacterial infections in livestock [1,2]. However, SDZ can lead to residues in animal-derived foods when overused. These residues can not only accumulate in humans through the food chain and cause serious health problems, but can also pollute soil or water by the pathway of animal excretion, leading to ecotoxicological contamination [3,4]. After discovering its harmful effects, the maximum residue level of SDZ has been set at 100 µg/kg in the EU, the USA, and China. Therefore, monitoring the residues of SDZ in food and the environment is of great significance [5,6]. In previous studies, SDZ was detected by HPLC, capillary electrophoresis, enzyme-linked immunosorbent assay, and time-resolved immunoassay [7,8,9,10,11]. Although these approaches are highly sensitive, the necessity of specialized operators, high costs, weak stability of antibodies, and the potential of cross-reactivity limit their application. Therefore, a simple, effective, and economical method is required to measure SDZ in food and environmental risk monitoring.

Aptamer development as a new type of recognition probe provides new ideas for the development of its detection. Aptamers are single-stranded DNA (ssDNA) or RNA that specifically bind to target molecules. Aptamers have superior stability, excellent affinity, strong specificity, and easy in vitro screening compared to antibodies [12,13,14]. With these advantages, various aptamer-based sensors have been widely developed for environmental monitoring and food safety inspection (e.g., fluorescent, electrochemical, surface-enhanced Raman scattering, and photoelectrochemical sensing signals) [15,16,17]. In comparison, fluorescent aptasensors have been widely used in the field of detection for their ability of sensitivity, low cost, and selectivity, which make them particularly useful in monitoring food and environmental samples [18,19], and fluorescent aptamer sensors based on nanomaterials (quantum dots, graphene oxide, nanosilicon, etc.) have attracted considerable attraction [20,21,22]. In particular, manganese dioxide (MnO_2_) has attracted much attention for its low cost, strong molar extinction coefficient, environmental tolerance, and non-toxicity, which has been widely used in biomedical and biosensing fields [22,23]. As a constantly developing two-dimensional nanomaterial, MnO_2_ nanosheets are easy to prepare on a large scale and possess broad absorption peaks that can overlap with the emission spectra of many fluorescent materials, making them considered to be quencher candidates. In addition, because MnO_2_ has a large specific surface area and good biocompatibility, it can greatly improve the detection of targets. Xu et al. reported that MnO_2_ nanosheets adsorb ssDNA through van der Waals forces interacting with bases, thus exhibiting strong absorption and efficiency of fluorescence burst [24]. Consequently, Qin et al. established an upconversion MnO_2_-based biosensor for the detection of carbendazim pesticides in food [25]. Li et al. constructed an open fluorescence sensor based on carbon dot-labeled oligodeoxyribonucleotide and MnO_2_ nanosheets for the detection of mercury(II) [26]. However, MnO_2_-based fluorescent aptasensors for the detection of SDZ residues have not been reported so far.

In this research, we constructed an aptasensor for selective detection of SDZ using FAM-labeled aptamer (FAM-SDZ30-1) and MnO_2_ nanosheets as donor-acceptor pair for the first time. Molecular dynamics simulations were performed to study the reason for the high specificity for SDZ by SDZ30-1. The performance of the established aptasensor was tested in food and environmental samples by adding different concentrations of SDZ to five samples, including soil, lake water, river water, egg, and beef. Finally, we compared the results of this method with HPLC and explored their correlation, demonstrating that the aptasensor can detect SDZ in a variety of samples.

## 2. Materials and Methods

### 2.1. Chemicals and Materials

The SDZ 30-1 (5′-AACCCAATGGGAT-3′, K_d_ = 65.72 nM) was selected in our laboratory, which was purified using HPLC and synthesized by Sangon Biotech, Shanghai, China [24]. All antibiotics, e.g., SDZ, tetracycline (TC), furaltadone (FTD), and norfloxacin (OFL) were bought from Sigma-Aldrich, St. Louis, MO, USA. KMnO_4_, hexadecyl trimethyl ammonium bromide (CTAB), buffer, and other reagents were purchased from Aladdin Co., Ltd., Ontario, CA, USA. The accuracy of the method was verified by HPLC on LC-20A (Shimadzu, Kyoto, Japan). Fluorescence intensity measurements were performed at the excitation wavelength of 492 nm and emission wavelength of 518 nm using Varioskan LUX.

### 2.2. Synthesis of MnO_2_

The MnO_2_ nanosheets were prepared as previously described [23]. Specifically, 0.5 g KMnO_4_ was dissolved in 450 mL of water and stirred for 30 min, then 1.5 g of CATB was added to form a stable emulsion. Next, 50 mL of 0.1 M MES (pH 6.0) was added to the above mixture and stirred for 12 h until a black color was formed. The product was centrifuged at 8000 rpm for 10 min and then washed thrice alternately with water and alcohol. Finally, the collected product was dried under a vacuum at 60 °C for 12 h. MnO_2_ was characterized by transmission electron microscope images (TEM), the UV-Vis, EDS elemental mapping and Fourier transform infrared spectrometer (FTIR).

### 2.3. Molecular Dynamics Simulation

The interactions between aptamers and different targets were compared through molecular dynamics simulations [27,28]. Firstly, the structures and parameters of the aptamer and target molecules were obtained from SwissParam. Subsequently, implicit solvation simulations were performed for the aptamer to target under constant temperature, volume, pressure, etc., while the obtained structures were subjected to an explicit solvent model of molecular dynamics simulations. Finally, the stability was measured using the root mean squared deviation (RMSD) produced by the molecular dynamics simulations trajectory. When the RMSD curve showed a slope upward tendency, the conformation of the system might undergo significant movement, and the smooth oscillation of the RMSD curve around a certain height indicated that the system had reached equilibrium. Other than that, a lower RMSD value means that the structural deviation of the binomial has small and higher stability.

### 2.4. Optimization of Aptasensor Conditions 

We optimized the detection conditions to obtain high sensitivity and selectivity with the established aptasensor. First, to determine the optimal concentration of FAM-SDZ30-1 with MnO_2_, different concentrations of MnO_2_ (0, 25, 50, 75, 100, 125, 150, 175, 200 μg/mL) were incubated with 50 nM aptamer. After centrifugation, the fluorescence intensity of the supernatant was measured, and the optimal concentration of MnO_2_ was determined. Subsequently, at the optimal MnO_2_ concentration, different concentrations of aptamer (50, 100, 200, 400, 600, 800 nM) were incubated with 100 ng/mL SDZ, followed by the addition of MnO_2_ to the mixture, incubation, and centrifugation to determine the optimal aptamer concentration based on fluorescence intensity. To identify the optimal reaction time of this sensor, MnO_2_ was added to the FAM-SDZ30-1 and incubated for 0, 2, 4, 6, 8, 10, 12, and 14 min, and the optimal time of the aptamer with MnO_2_ was determined by the value of fluorescence. To find the effects of the reaction time of SDZ with the FAM-aptamer on the fluorescence intensity, the aptamer was then incubated with SDZ for 10, 20, 30, 40, 50, and 60 min. The fluorescence intensity of the supernatant was measured, and the best incubation time of the aptamer with the target was determined. Finally, to explore the effects of the binding buffer on the aptasensor, 400 nM of FAM-SDZ30-1 was incubated with 100 ng/mL of SDZ with four different buffers (HEPES, TE, PBS, Tris-HCl), followed by the addition of 150 μg/mL of MnO_2_, incubated and centrifuged, resulting in the determination of the optimized buffer according to the fluorescence in the supernatant. Similarly, the aptasensor was kept at different pH values (6.4, 6.9, 7.4, 7.9, 8.4) and operated as described above to determine the optimal pH based on the fluorescence intensity.

### 2.5. Standard Curves of the Aptasensor

Briefly, 40 μL of FAM-labeled aptamer (2 μM) was mixed with SDZ at various concentrations (5, 10, 20, 40, 80, 100, 200, and 400 ng/mL) after gentle shaking for 50 min at 25 °C in the dark. After that, 30 μL MnO_2_ (1 mg/mL) was added, and the mixture was incubated for 10 min and centrifuged at 10,000 rpm for 5 min. The fluorescence intensity of the supernatant was measured and a standard curve was established with different SDZ concentrations. The limit of detection (LOD) was acquired by calculating the 3SD/slope (the slope of the linear curve), and the SD was the standard deviation from the fluorescence values of a blank sample [29].

### 2.6. Determination of Selectivity

The selectivity was evaluated by adding 10 µL (100 ng/mL) of different structurally related compounds, e.g., sulfaquinoxaline (SQX), sulfamethazine (SMZ), tetracycline (TC), chlortetracycline (CTC), nitrofurazone (NFZ), furaltadone (FTD), norfloxacin (NOR) dissolved in 400 nM FAM-SDZ30-1 for 50 min to individual aptasensor against SDZ. After centrifugation, the fluorescence intensity was measured in the supernatant. After taking measurements for different antibiotics with relative fluorescence intensity F and the blank sample with fluorescence intensity F_0_, the ∆F (∆F = F − F_0_) was calculated [30].

### 2.7. Sample Preparation

All samples were prepared as previously described with slight modifications [31]. To prepare soil samples, 2 g of soil was diluted 10 times with PBS (pH 7.4), centrifuged at 13,000 rpm for 20 min, and filtered through a 0.22 µm filter membrane. Lake and river water samples were prepared and diluted 10 times as described above; 2 g of eggs were completely mixed with 4 mL of ethyl acetate and vortexed for 10 min, the supernatant after centrifugation was evaporated under a stream of nitrogen at 40 °C to remove the ethyl acetate from the mixture and finally dissolved in the buffer. Next, 5 g beef was homogenized in a homogenizer, then 25 mL of acetonitrile was added, and the mixture was vortexed and shaken for 15 min, ultrasonicated for 10 min, and then centrifuged at 12,000 rpm for 15 min. The supernatant was moved to 30 mL of acetonitrile-saturated hexane and constantly stirred for 10 min to clear the fat. The organic solvent was removed at 80 °C in a water bath, and the residue was dissolved in 5 mL of binding buffer, diluted 10 times, and filtered through a membrane.

### 2.8. Assay Validation 

Five different samples were used to validate the properties of the aptasensor. First, HPLC was used to confirm that all samples did not have SDZ, and then SDZ was added to the processed sample buffer. The standard curves of the sample matrices were constructed according to the aptasensor method described above. The precision and accuracy of the fluorescent aptasensor were verified by analyzing the samples mentioned above with different SDZ concentrations (10, 20, 30 µg/mL), testing five times for each concentration. To test the credibility of the aptasensor in food and environmental samples, the results of the aptasensor and HPLC were compared using the same samples. Following the established procedure, the HPLC analysis was performed on all spiked samples containing different SDZ concentrations. The linear regression was used to calculate the correlation between the results of aptasensor and HPLC. Recoveries were calculated as (measured concentration/known concentration) × 100%, and the coefficient of variation was determined [32]. Each spiked sample was analyzed five times.

## 3. Results and Discussion 

### 3.1. Principle of the Aptasensor

**Figure 1:**
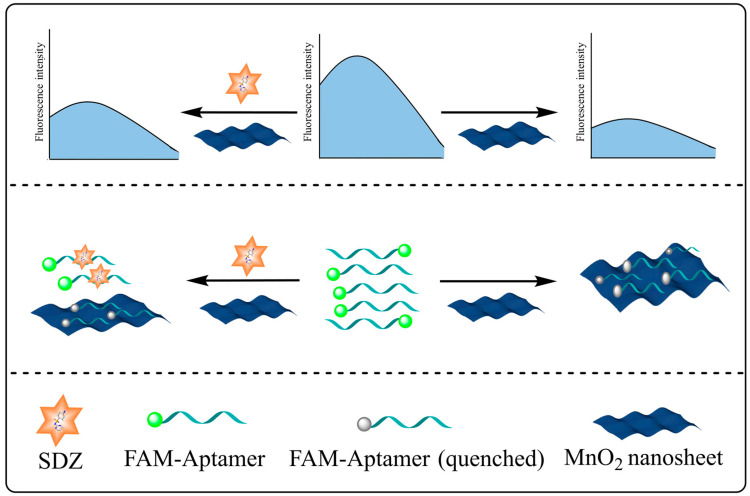
Schematic diagram of the aptasensor based on MnO_2_ for the detection of SDZ.

The FAM-SDZ30-1 fluorescence was quenched by MnO_2_ through π-π stacking, electrostatic adsorption, and electron-induced transfer in the absence of SDZ30-1 (Figure 1). In contrast, in the presence of SDZ, FAM-SDZ30-1 selectively combines with SDZ instead of being adsorbed by MnO_2_, preventing FAM-SDZ30-1 fluorescence quenching.

**Figure 2:**
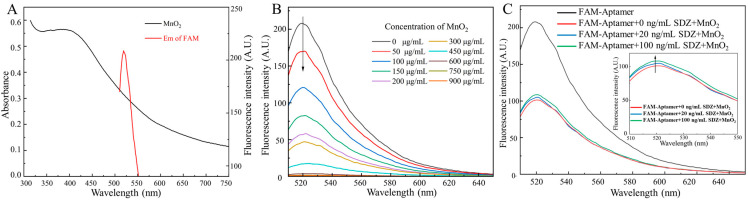
(**A**) UV-Vis absorption spectra of MnO_2_ and fluorescence emission spectra of FAM. (**B**) Effects of different MnO_2_ concentrations on the fluorescence spectra of FAM. (**C**) Fluorescence intensity of FAM in different reaction systems.

Specifically, the absorption spectrum of MnO_2_ and the fluorescence emission spectrum of FAM overlap as MnO_2_ is near FAM-SDZ30-1, resulting in FRET (Figure 2A). With the increase of the concentrations of MnO_2_, the fluorescence intensity of the aptamer continuously decreased. When the concentration of MnO_2_ was extremely high, the fluorescence intensity of the aptamer tended to 0, indicating that the fluorescence of the aptamer was completely quenched (Figure 2B). The fluorescence intensity of FAM in different reaction systems is shown in Figure 2C. When only the aptamer was in the system, the fluorescence intensity was the highest, and after the addition of MnO_2_, it will FRET with the aptamer to decrease the fluorescence value. When the target was added to the system, the aptamer and the target could bind specifically, recovering the fluorescence. Moreover, the higher concentration of SDZ, the stronger value of fluorescence was detected. Hence, the fluorescent aptasensor for SDZ detection was constructed based on the correlation between SDZ concentration and fluorescence values.

### 3.2. Characterization of MnO_2_

**Figure 3:**
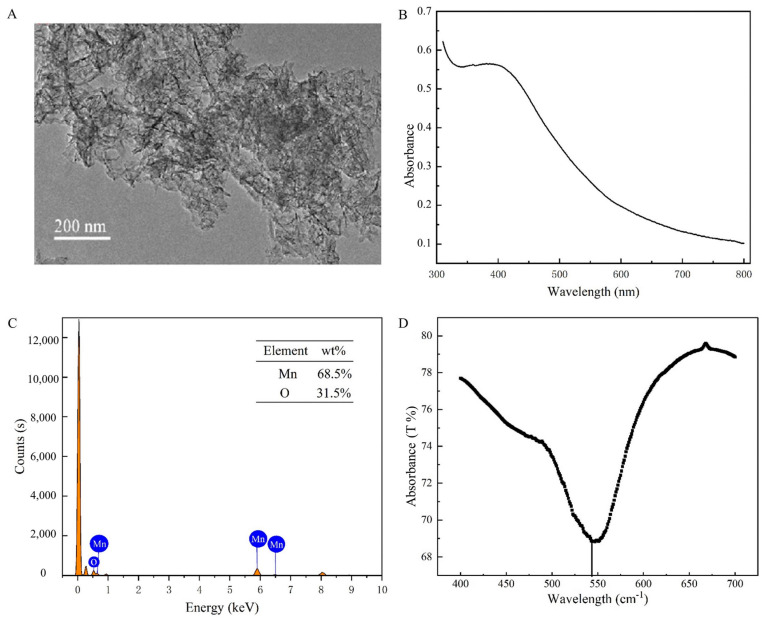
(**A**) TEM images of MnO_2_. (**B**) The UV−Vis of MnO_2_. (**C**) EDS elemental mapping of MnO_2_. (**D**) The FTIR of MnO_2_.

The TEM images showed that the synthesized MnO_2_ nanosheets showed a homogeneous structure with a diameter of about 200 nm (Figure 3A). The UV-Vis showed the absorption peak of MnO_2_ from 300 to 600 nm (Figure 3B), and the EDS elemental mapping showed that Mn accounted for 68.5% and O 31.5%, demonstrating the successful preparation (Figure 3C). In the MnO_2_ curves (Figure 3D), the peaks appearing at 500 to 600 cm^−1^ correspond to Mn-O bending vibration peaks [23]. These results indicated that MnO_2_ nanosheets were successfully synthesized.

### 3.3. Molecular Simulation Analysis

**Figure 4:**
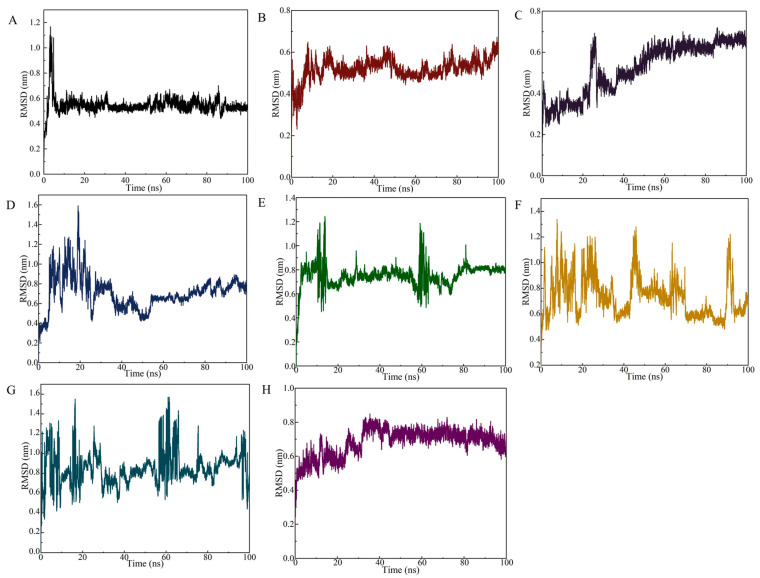
RMSD of the SDZ30-1 aptamer against SDZ and other antibiotics. (**A**) SDZ; (**B**) SMZ; (**C**) SQX; (**D**) tetracycline; (**E**) norfloxacin; (**F**) chlortetracycline; (**G**) nitrofurazone; (**H**) furaltadone.

The RMSD curve of the SDZ/SDZ30-1 complex increased slowly from 0 to 15 ns (Figure 4A), indicating that the backbone of the key nucleic acid was moving during this time. Between 15 ns and 100 ns, the complex equilibrated and the RMSD curve fluctuated only around 0.6 Å, with the amplitude remaining within 0.2 Å. In the 0–100 ns range, the RMSD values of the molecular dynamics simulations of the aptamer with other target systems fluctuated strongly, indicating that SDZ30-1 was violently moving with other antibiotics and forming destabilized complexes (Figure 4B–H). Specifically, although SDZ30-1/SMZ reached relative stability at around 20 ns, the amplitude was observed to be large, probably because of their structural similarity. SDZ30-1 with SQX only arrived at relative stability at around 50 ns, yet it continuously increased after 60 ns. The complex formed by SDZ30-1/TC and SDZ30-1/OFL was not balanced until 35 ns the RMSD curve fluctuated around 0.1 Å. Although the complex formed by SDZ30-1 with CTC reached equilibrium at 15 ns, the RMSD curve moved again at about 60 ns. Furthermore, it was evident from the RMSD curves that SDZ30-1/NFZ and SDZ30-1/FTD were not in equivalent equilibrium between 0 and 100 ns, probably because they belong to different antibiotic categories. These results suggested that the aptamer has a high selectivity for SDZ.

### 3.4. Optimization of the Aptasensor

**Figure 5:**
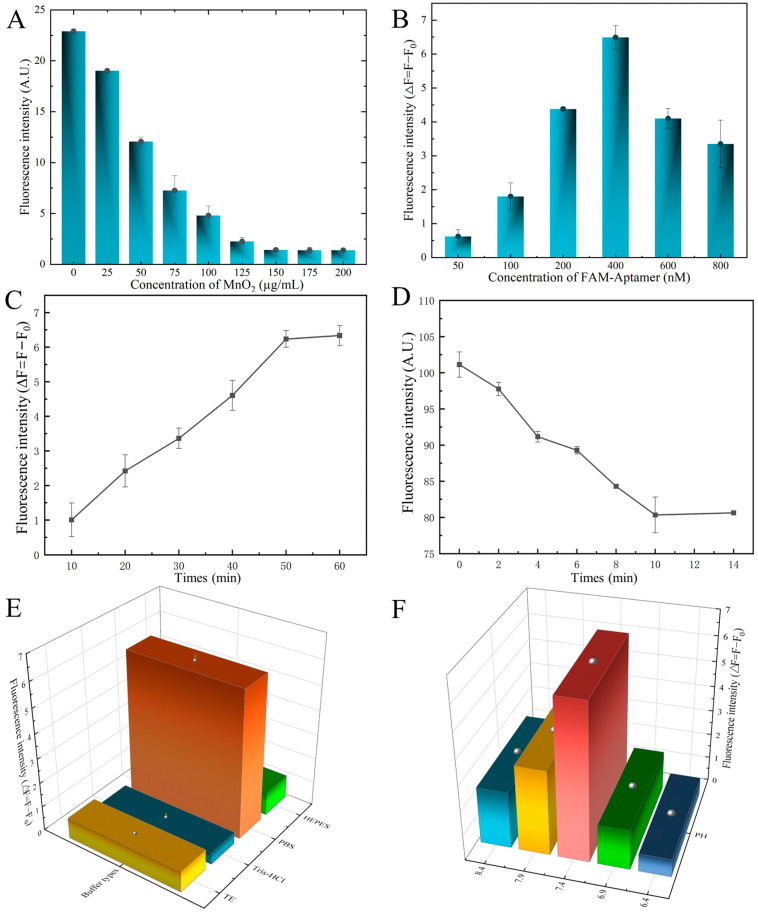
Optimization of the aptasensor system. (**A**) Concentration of MnO_2_. (**B**) Concentration of aptamer. (**C**) Incubate time of aptamer with MnO_2_. (**D**) Incubate time of aptamer with SDZ. (**E**) Optimization of binding buffer. (**F**) Optimization of pH.

Moreover, we found that as the concentration between FAM-SDZ30-1, MnO_2,_ and SDZ was optimized, the higher the concentration of MnO_2_; the lower the fluorescence intensity of the system, when the concentration reached 150 µg/mL, the fluorescence intensity stabilized, and thus, 150 µg/mL was chosen as the best MnO_2_ concentration (Figure 5A). When the concentration of aptamer was 400 nM, the fluorescence value of the system was the highest, and therefore 400 nM was taken as the optimal concentration of the aptamer (Figure 5B). The fluorescence intensity of the aptamer and SDZ reached stability as the time up to 50 min (Figure 5C), thus 50 min was the best incubation time. The fluorescence intensity decreased gradually with the increase of time, and the fluorescence stabilized when the time arrived at 10 min, thereby selected as the best quenching time (Figure 5D). The highest fluorescence intensity of this system was also observed in the PBS buffer (Figure 5E). The pH value affected the fluorescence intensity of the fluorescent aptasensor, and 7.4 was the best pH condition (Figure 5F).

### 3.5. Properties of Aptasensor

**Figure 6:**
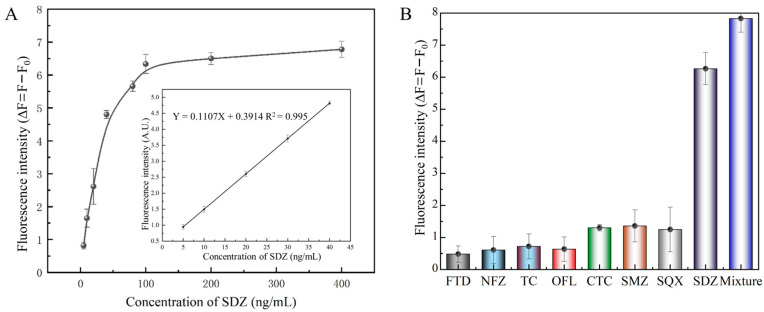
Properties of the fluorescent aptasensor. (**A**) Standard curve of the aptasensor. (**B**) Specificity of SDZ30-1 against SDZ and other antibiotics.

The linear range for SDZ detection varied from 5 to 40 ng/mL with a LOD of 3.25 ng/mL (Figure 6A). Then, we compared this performance to several reported SDZ detection methods. The developed fluorescent aptasensor displayed a similar linear range to traditional methods, such as HPLC, UPLC-MS/MS, and ELISA (Table 1). Compared to electrochemical biosensors, the proposed fluorescence aptasensor avoids complex electrode modifications and is relatively simple to operate. Then, similar fluorescent biosensors based on nanomaterials were compared. The linear range and LOD of the fluorescent biosensor constructed in this study were similar to several previous methods. 

Meanwhile, in order to assess the detection selectivity for SDZ of this aptasensor, SDZ, and other antibiotics were detected using the FRET-based aptasensor. The fluorescence intensity of SDZ30-1 bound to all other antibiotics was relatively slow (Figure 6B). Specifically, the fluorescence intensity of SDZ30-1 was similar to SMZ and SQX, probably because they belong to the family of sulfonamide antibiotics and have similar structures. The fluorescence values of SDZ30-1 with FTD, NFZ, TC, and OFL were very low, indicating that the aptasensor has high specificity. Taken together, these results indicated that this aptasensor has excellent selectivity.

### 3.6. Validation of the Aptasensor

**Figure 7:**
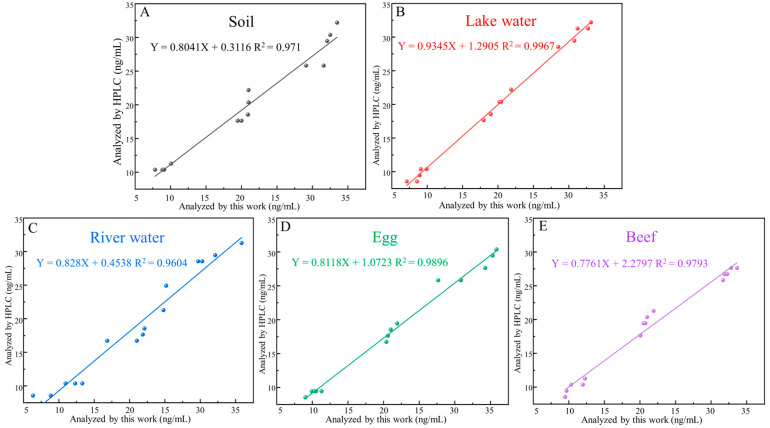
Standard curves corresponding to detection in food matrix, including (**A**) soil, (**B**) lake water, (**C**) river water, (**D**) egg, (**E**) beef.

Different SDZ concentrations (10, 20, and 30 ng/mL) were analyzed in spiked samples to demonstrate the properties of the aptasensor in highly complex biologic samples. The average recovery of these samples was between 87.19% and 109.26%, and the coefficients of variation were between 3.13% and 13.14% (Table 2). Furthermore, the sensor showed a positive relationship with its HPLC (Figure 7), demonstrating that it can reliably detect SDZ in animal-derived foods and the environment. Overall, the simple synthesis process and better burst performance of MnO_2_ nanosheets make the developed FRET-based aptasensor simpler and less expensive, showing the promising potential of the method for application in real samples.

## 4. Conclusions

In summary, we first developed a fluorescent aptasensor based on FAM-SDZ30-1 and MnO_2_ to detect SDZ in food and the environment. The molecular dynamics simulations showed that RMSD values fluctuated from 10 to 100 ns around 0.6 nm in the SDZ30-1 and SDZ systems, indicating the high selectivity of SDZ30-1. The linear detection range (5–40 ng/mL) and LOD (3.25 ng/mL) of the aptasensor were sensitive to SDZ detection. The specificity of this aptasensor was satisfactory compared to various antibiotics. Additionally, the correlation between the aptasensor and HPLC was high (R^2^ ≥ 0.9604), demonstrating its precision in detecting SDZ. Therefore, this fluorescent aptasensor is simple to operate, low cost, and selective, and can be used as a reliable tool to monitor SDZ in different samples with promising applications in detection.

## Figures and Tables

**Table 1:** Comparison of the method proposed in this study with other methods previously for SDZ.

Method	LOD (ng/mL)	References
HPLC	0.9	Alipanahpour et al. (2021) [8]
Spectrophotometry	19	Errayess et al. (2017) [9]
Chitosan-based ELISA	8.64	Zeng et al. (2021) [10]
Electrochemical	2.25	Kokulnathan et al. (2021) [2]
Aptasensor based on gold nanoparticles	2	Yang et al. (2022) [32]
Aptasensor based on MnO_2_	3.25	Present work

**Table 2:** Mean recoveries and coefficients of variation in spiked samples (*n* = 5).

Sample	Spiked (µg/kg)	This Work			HPLC		
		Found (µg/kg)	Recovery (%)	CV (%)	Found (µg/kg)	Recovery (%)	CV (%)
Soil	10	8.96	89.58	12.71	10.55	105.45	3.39
	20	20.47	102.36	8.03	19.27	96.36	9.51
	30	31.75	105.84	7.45	28.73	95.76	9.38
Lake water	10	8.72	87.19	11.21	9.45	94.55	8.33
	20	19.88	99.39	7.81	19.82	99.09	8.33
	30	31.27	104.23	9.98	30.55	101.82	4.67
River water	10	10.35	98.71	11.99	9.64	96.36	8.98
	20	21.33	106.65	9.28	18.18	90.91	9.60
	30	30.62	102.07	3.13	28.55	95.15	7.73
Egg	10	10.22	102.18	7.74	9.27	92.73	3.79
	20	20.90	104.50	7.66	18.00	90.00	5.33
	30	32.78	109.26	13.14	27.82	92.73	7.08
Beef	10	10.74	107.39	9.10	10.00	100.00	9.05
	20	20.85	104.23	7.16	19.64	98.18	6.39
	30	32.46	108.19	5.49	26.91	89.70	2.68

## Data Availability

Not applicable.
